# NFAT5 Restricts Bovine Herpesvirus 1 Productive Infection in MDBK Cell Cultures

**DOI:** 10.1128/spectrum.00117-23

**Published:** 2023-05-25

**Authors:** Chang Liu, Jiayu Lin, Hao Yang, Ningxi Li, Linke Tang, Donna Neumann, Xiuyan Ding, Liqian Zhu

**Affiliations:** a College of Life Sciences, Hebei University, Baoding, China; b Department of Ophthalmology and Visual Sciences, University of Wisconsin-Madison, Madison, Wisconsin, USA; c Key Laboratory of Microbial Diversity Research and Application of Hebei Province, College of Life Science, Hebei University, Baoding, China; Oklahoma State University College of Veterinary Medicine

**Keywords:** NFAT5, BoHV-1, mitochondria

## Abstract

Bovine herpesvirus 1 (BoHV-1), an important bovine viral pathogen, causes severe disease in the upper respiratory tract and reproductive system. Tonicity-responsive enhancer-binding protein (TonEBP), also known as nuclear factor of activated T cells 5 (NFAT5), is a pleiotropic stress protein involved in a range of cellular processes. In this study, we showed that the knockdown of NFAT5 by siRNA increased BoHV-1 productive infection and overexpression of NFAT5 via plasmid transfection decreased virus production in bovine kidney (MDBK) cells. Virus productive infection at later stages significantly increased transcription of NFAT5 but not appreciably alter measurable NFAT5 protein levels. Virus infection relocalized NFAT5 protein and decreased the cytosol accumulation. Importantly, we found a subset of NFAT5 resides in mitochondria, and virus infection led to the depletion of mitochondrial NFAT5. In addition to full-length NFAT5, another two isoforms with distinct molecular weights were exclusively detected in the nucleus, where the accumulation was differentially affected following virus infection. In addition, virus infection differentially altered mRNA levels of PGK1, SMIT, and BGT-1, the canonical downstream targets regulated by NFAT5. Taken together, NFAT5 is a potential host factor that restricts BoHV-1 productive infection, and virus infection hijacks NFAT5 signaling transduction by relocalization of NFAT5 molecules in cytoplasm, nucleus, and mitochondria, as well as altered expression of its downstream targets.

**IMPORTANCE** Accumulating studies have revealed that NFAT5 regulates disease development due to infection of numerous viruses, underlying the importance of the host factor in virus pathogenesis. Here, we report that NFAT5 has capacity to restrict BoHV-1 productive infection *in vitro*. And virus productive infection at later stages may alter NFAT5 signaling pathway as observed by relocalization of NFAT5 protein, reduced accumulation of NFAT5 in cytosol, and differential expression of NFAT5 downstream targets. Importantly, for the first time, we found that a subset of NFAT5 resides in mitochondria, implying that NFAT5 may regulate mitochondrial functions, which will extend our knowledge on NFAT5 biological activities. Moreover, we found two NFAT5 isoforms with distinct molecular weights were exclusively detected in the nucleus, where the accumulation was differentially affected following virus infection, representing a novel regulation mechanism on NFAT5 function in response to BoHV-1infection.

## INTRODUCTION

Bovine herpesvirus type 1 (BoHV-1) is a member of the family *Herpesviridae* and the subfamily *Alphaherpesvirinae* (1). As an important pathogen for cattle, the virus infection causes a severe inflammatory response in the upper respiratory tract, which may lead to a secondary infection by other pathogens, such as bovine viral diarrhea viruses (BVDV), bovine respiratory syncytial virus (BRSV), parainfuenza-3 virus (PI3V), bovine coronaviruses, Mannheimia haemolytica, Pasteurella multocida, Histophilus somni, and *Mycoplasma* spp. These could ultimately lead to bovine respiratory disease complex (BRDC), a life-threatening pneumonia (2). Thus, BoHV-1 is a cofactor in the development of BRDC [2, 3]. In addition, BoHV-1 is an important pathogen closely associated with viral abortion storms in the cattle industry in North America (3, 4). As one of the most important diseases in cattle, virus infection and pathogenesis causes great economic loss to the cattle industry worldwide.

Nuclear factor of activated T cells 5 (NFAT5), also referred to as tonicity-responsive enhancer-binding protein (TonEBP), was initially identified as a hypertonic (high salt) stress-sensitive transcriptional regulator in the renal medulla (5, 6). Mechanically, hypertonic conditions activate NFAT5, which may lead to cytoplasm-nucleus translocation of the NFAT5 protein (7–9). In the nucleus, NFAT5 binds to and stimulates the promoter region of target genes responsible for osmoregulation, such as aldose reductase (AR), taurine transport protein (TauT), Na+-dependent myo-inositol cotransporter (SMIT), and betaine/γ-aminobutyric acid transporter (BGT1) (10). Subsequent studies indicated that NFAT5 was a ubiquitous transcription factor widely expressed in various tissues with pleiotropic functions, such as embryonic development, and cell differentiation (11, 12). As an important regulator of both innate and adaptive immunity, NFAT5 is also implicated in the pathogenesis of various diseases, including various inflammatory diseases and cancer development (13–18).

Accumulating literatures have revealed a panel of molecules interacting with NFAT5. For instance, it has been reported that high-salt conditions activate the p38/MAPK pathway involving NFAT5 and serum/glucocorticoid-regulated kinase 1 (SGK1) during cytokine-induced TH17 polarization (19). NFAT5 binds to the β-catenin transactivation C-terminal domain and prevents CBP interaction with β-catenin, thus inhibiting β-catenin acetylation and Wnt/β-catenin activation (20). Of note, p38/MAPK (21, 22), SGK1 (23), and β-catenin (24) are involved in the virus productive infection, particularly at the later stages of lytic infection, further suggesting that NFAT5 may also be involved in the BoHV-1 productive infection at later stages.

To further evaluate this hypothesis, we investigated the interplay between NFAT5 and BoHV-1 productive infection. Interestingly, for the first time, we found that NFAT5 restricts BoHV-1 productive infection, and virus infection affects NFAT5 signaling transduction *via* various approaches. These results are outlined below.

## RESULTS

### BoHV-1 productive infection relocalized NFAT5 proteins.

**FIG 1:**
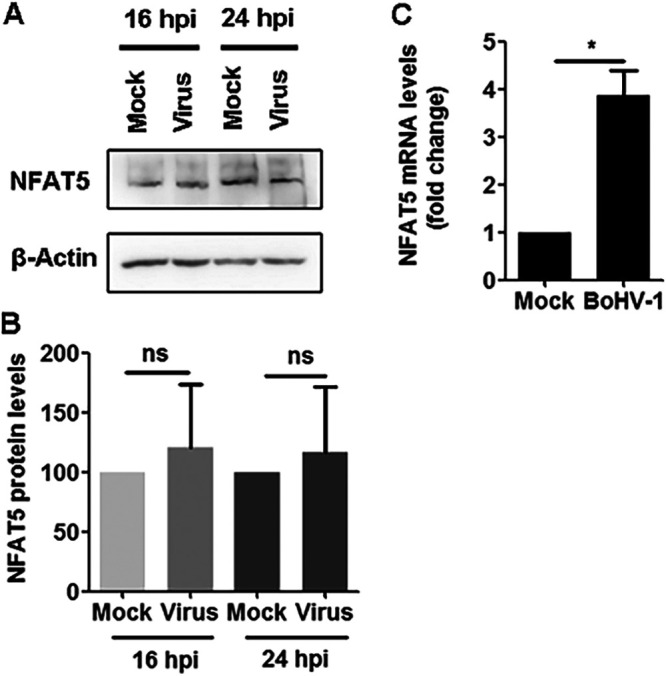
BoHV-1 infection has no obvious effects on NFAT5 steady-state protein levels at late stages after infection. (A) Confluent MDBK cells in 60 mm dishes were infected with BoHV-1 at an MOI of 0.1 for 16 h and 24 h (h). Cells were lysed with RIPA buffer and then analyzed by Western blotting to detect NFAT5 protein levels. β-Actin was detected as a loading control. (B) Band intensity was analyzed with free software Image J. Each analysis was compared with that of mock-infected controls at indicated times after infection, which was arbitrarily set as 1. (C) MDBK cells in 6-well plates were either mock-infected or infected with BoHV-1 at an MOI of 0.1. At 24 h postinfection, the total RNA was purified, and the mRNA levels of NFAT5 were examined with qRT-PCR. The results shown are representations of three independent experiments, with error bars indicating standard deviations. Significance was assessed with a Student's *t* test (*, *P *< 0.05; ns, not significant).

To understand whether the NFAT5 signaling affects BoHV-1 productive infection in cell culture, we initially examined NFAT5 protein levels following infection of bovine kidney (MDBK) cells at later stages. At 16 and 24 h after infection, NFAT5 protein levels were similar to mock-infected cells (Fig. 1A and B). At the same time, the virus infection increased NFAT5 transcription. Relative to the uninfected cells, mRNA levels of NFAT5 significantly increased by approximately 3.87-fold following virus infection (Fig. 1C). Therefore, the unaltered measurable NFAT5 protein levels do not correspond to the increased mRNA levels in response to virus infection.

**FIG 2:**
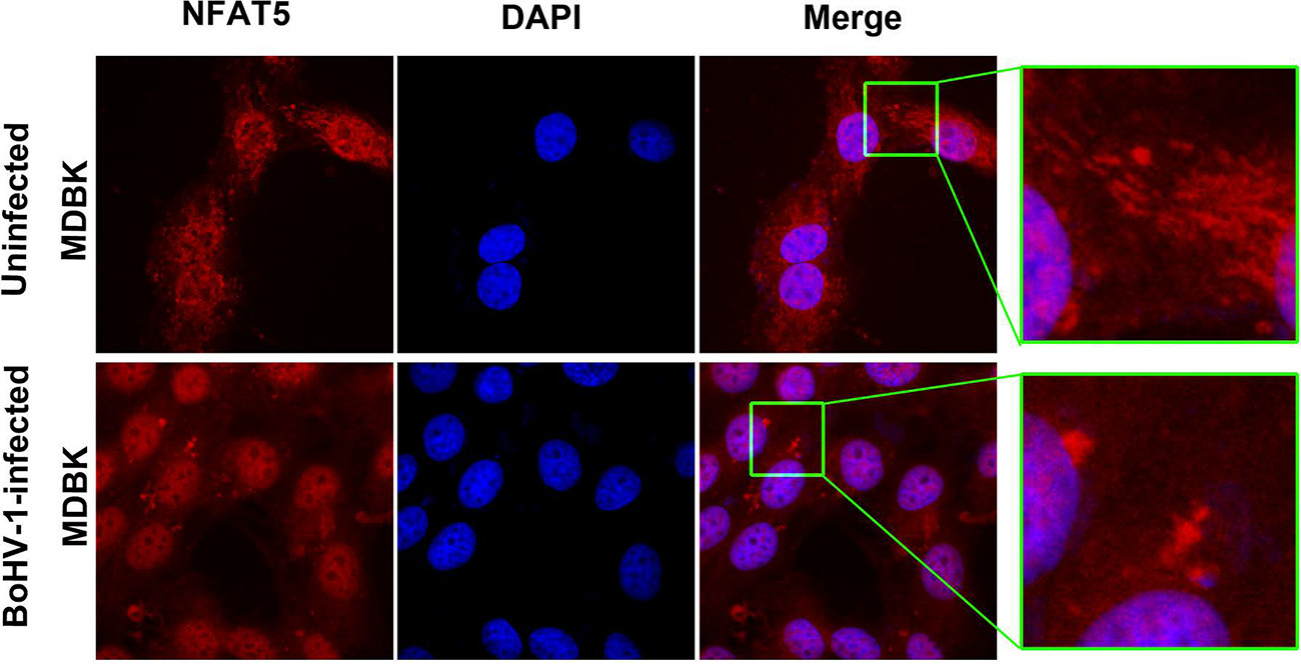
Effects of BoHV-1 infection had on NFAT5 localization. MDBK cells in a 2-well chamber slide were either mock-infected or infected with BoHV-1 at an MOI of 0.1. After infection for 24 h, the cells were fixed with 4% formaldehyde, and NFAT5 protein was immunostained by using NFAT5-specific MAb via IFA. DAPI staining was used to stain nuclear DNA. Images were obtained by performing confocal microscopy (Leica).

Because NFAT5 may have distinct biological functions at distinct subcellular locations in response to diverse stimulators, confocal microscopy was performed to assess whether NFAT5 localization was influenced at 24 h after infection. In mock-infected MDBK cells, NFAT5 was dispersed throughout the cells (Fig. 2). Interestingly, a large amount of speckle-like staining of highlighted immunofluorescence was readily observed in both cytoplasm and nucleus (Fig. 2, upper panels). Nuclear NFAT5 proteins were evenly dispersed following virus infection, with only a small amount of speckle-like staining left in the cytoplasm (Fig. 2, bottom panels). Notably, NFAT5 was rarely detected in the isolated fractions of the cell membrane (data not shown). The observed changes occurred in the cytosol and nucleus. These data suggested that virus infection relocalized NFAT5 proteins, a potential mechanism to alter NFAT5 signaling pathways.

### BoHV-1 productive infection altered the accumulation of NFAT5 proteins in mitochondria.

**FIG 3:**
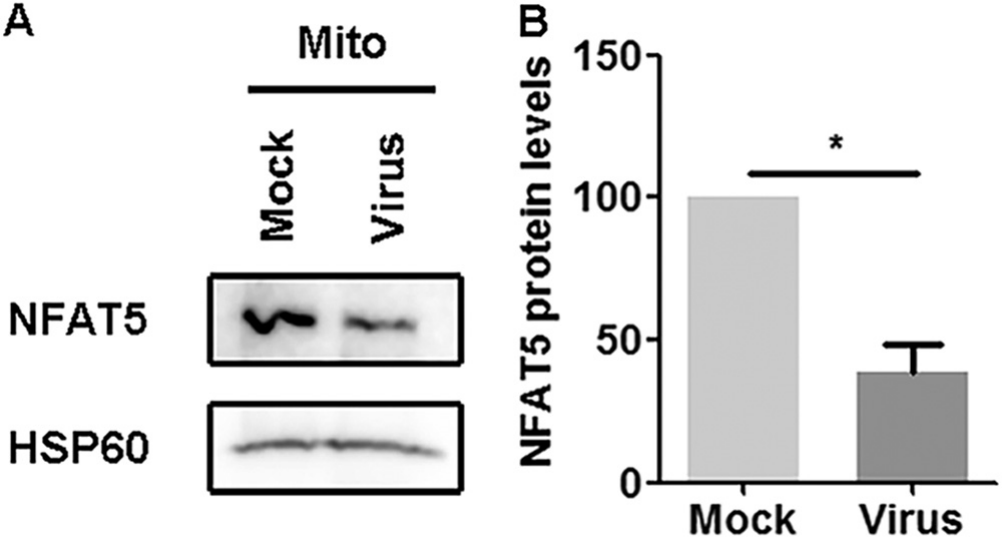
Effects of the BoHV-1 infection had on the accumulation of NFAT5 in mitochondria. (A) MDBK cells in 100 mm dishes were either mock-infected or infected with BoHV-1 (MOI = 0.1) for 24 h. The cells were collected for isolation of mitochondria fractions via using a commercial kit (Beyotime Biotechnology, cat# C3601) following the manufacturer’s protocol. Protein levels of NFAT5 were detected by Western blotting. HSP60 was detected and used as a protein loading control. (B) Band intensity was analyzed with the software Image J. The control was arbitrarily set as 1. The results shown are representations of three independent experiments. Error bars denote the variability between three independent experiments. Significance was assessed with a Student's *t* test (*, *P *< 0.05).

Immunofluorescence assays revealed a speckle-like staining pattern, particularly for cytoplasmic NFAT5 in the mock-infected cells (Fig. 2, upper panels), and we hypothesized that NFAT5 may be localized to mitochondria and altered by viral infection. To test this hypothesis, mitochondrial proteins were purified using a commercial kit (Beyotime Biotechnology, cat# C3601) specific for isolating mitochondria from cell cultures. Then the purified mitochondrial fractions were subjected to Western blot to detect NFAT5 protein. NFAT5 proteins were readily detected in mitochondria in mock-infected cells, and their protein levels were significantly reduced after viral infection (Fig. 3A). Relative to the mock-infected controls, NFAT5 protein levels were decreased by ~40% after viral infection (Fig. 3B), suggesting that virus infection decreased accumulation of NFAT5 in mitochondria.

### BoHV-1 productive infection altered nucleus accumulation of NFAT5 isoforms and transcription of its downstream targets.

**FIG 4:**
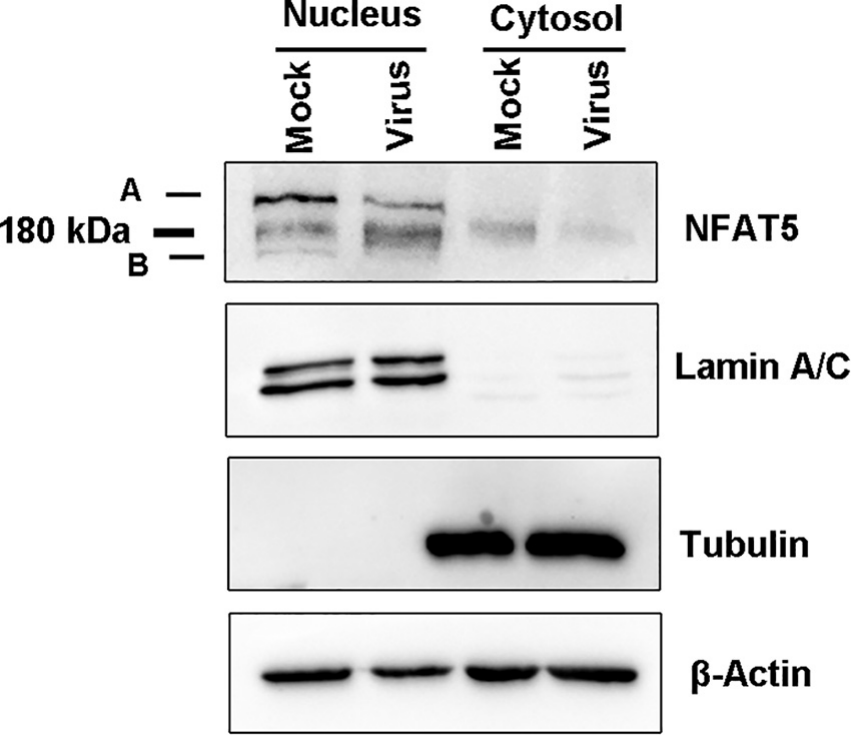
Effects of the BoHV-1 infection had on nuclear accumulation of NFAT5 proteins. (A) MDBK cells in 100 mm dishes were either mock-infected or infected with BoHV-1 (MOI = 0.1) for 24 h. The cells were collected for isolation of both nucleus and cytosol fractions via using a commercial kit (Beyotime Biotechnology, cat# P0027), following the manufacturer’s protocol. Protein levels of NFAT5 were detected by Western blotting. LaminA/C, a marker for nuclear protein, and β-tubulin, a marker for cytoplasmic protein, were detected as a protein loading control, respectively. The results shown are representations of three independent experiments.

Given that nuclear translocation of NFAT5 may enhance its transcriptional activity (25), we examined the effects that virus infection had on the accumulation of nuclear NFAT5 proteins. Cell cultures were either mock infected or virus infected for 24 h, then fractions of both cytosol and nucleus were isolated using a commercial nucleus isolation kit (Beyotime Biotechnology, cat# P0027), and subjected to Western blotting. Notably, full-length NFAT5 was expected to have a band at approximately 180 kDa. Here, we found there are three bands: one is exactly the expected band with a molecular weight of 180 kDa, one clear band with molecular weight of over 180 kDa (denoted by A), and a faint band with a molecular weight of less than 180 kDa (denoted by B). However, bands A and B were not readily detected in the cytosol fractions in either mock-infected or virus-infected cells. Relative to the mock-infected control, protein levels of both bands A and B were evidently decreased after virus infection. In contrast, the expected full-length NFAT5 was increased in the nucleus following virus infection. In addition, the protein levels of major NFAT5 bands were decreased in cytosol fractions attributed to virus infection (Fig. 4). LaminA/C, a marker for nuclear protein, was not detected in the cytoplasmic fraction, and β-tubulin, a marker for cytoplasmic protein, was not detected in the nuclear fractions, suggesting that neither fraction is contaminated by the counterpart (Fig. 4), validating the findings that virus infection promotes depletion of cytosol NFAT5 but differentially influenced accumulation of nuclear NFAT5 of different isoforms.

**FIG 5:**
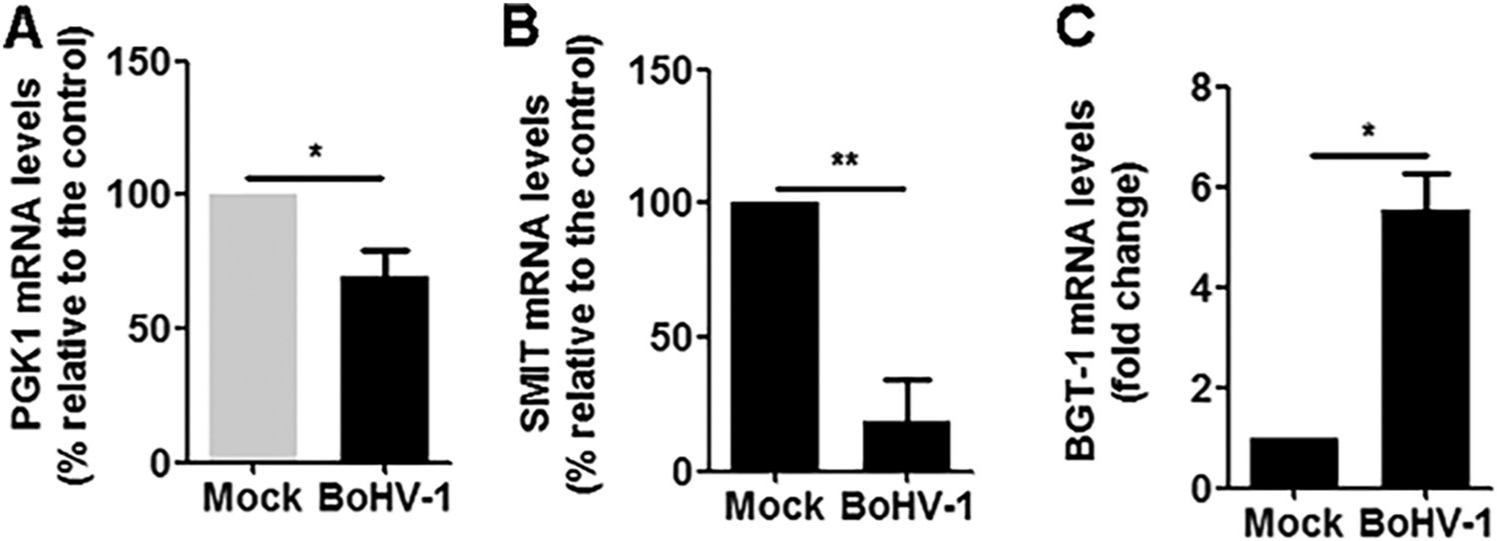
Effects of the BoHV-1 infection had on the transcription of NFAT5 downstream targets. MDBK cells in 6-well plates were either mock-infected or infected with BoHV-1 at an MOI of 0.1. At 24 h postinfection, total RNA was purified, and the mRNA levels of PGK1 (A), SMIT (B), and BGT-1 (C) were examined with qRT-PCR. The results shown are representations of three independent experiments, with error bars indicating standard deviations. Significance was assessed with a Student's *t* test (*, *P *< 0.05).

Because virus infection not only has an influence on the nuclear accumulation of NFAT5 protein but also relocalized nuclear NFAT5 proteins, we wondered whether the activity of NFAT5 protein was therefore influenced. For this purpose, we detected the mRNA levels of PGK1, SMIT, and BGT-1, the canonical downstream targets regulated by NFAT5 signaling. Our data indicated that the virus infection altered the transcription of these molecules in diverse manners: PGK1 and SMIT were decreased to 70% and 18%, respectively (Fig. 5 A and B), BGT-1mRNA levels were increased to approximately 5.54-fold (Fig. 5C), following virus infection. Obviously, the expression of these NFAT5-regulated genes appeared to differentially change in unexpected ways, which may suggest that their expression was impacted by other host or virus factors during infection.

### NFAT5 restricts virus productive infection.

**FIG 6:**
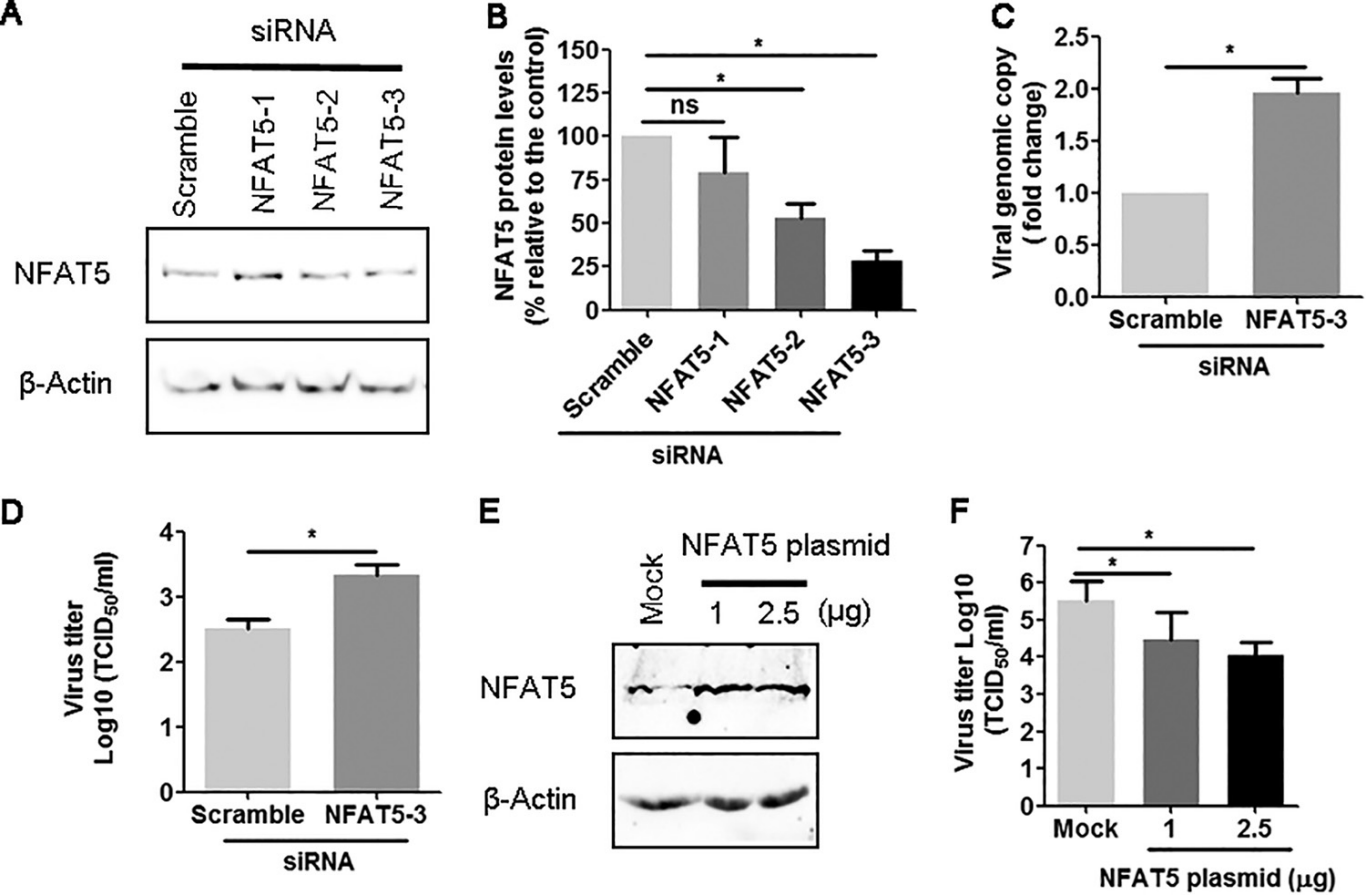
NFAT5-specific siRNA increases virus production in MDBK cells. (A) MDBK cells in 6-well plates were transfected with either scrambled siRNA (200 pmol) or three individual siRNA targeting NFAT5 (200 pmol), referred to as siRNANFAT5-1, siRNANFAT5-2, and siRNANFAT5-3, respectively. At 48 h after transfection, the effective siRNA were screened by detection of NFAT5 protein levels via Western blot. (B) Band intensity was analyzed with the software Image J. The control was arbitrarily set as 100%. (C and D) MDBK cells in six-well plates were transfected with either scrambled siRNA (200 pmol) or siRNANFAT5-3 (200 pmol). After transfection for 48 h, cell cultures were infected with BoHV-1 (MOI = 0.1). After infection for 24 h, the cellular genome was purified by using a commercial viral DNA purification kit, and the viral genome was examined by using qPCR using gC-specific primer(C). In parallel, cell infection virus yield in supernatants was examined with results expressed as TCID_50_/mL (D). (E and F) NFAT5 plasmid of 1 and 2.5 μg along with empty vector was transfected into MDBK cells in 6-well plates using, after transfection for 48 h, the cells were either collected for the detection of NFAT5 protein levels using Western blot (E), or subjected to virus infection (MOI = 0.1), and the virus production were tittered in MDBK cells with results expressed as TCID_50_/mL (F). The data represented three independent experiments. Results are the mean of three independent experiments, with error bars showing standard deviations. Significance was assessed with a Student's *t* test (*, *P*< 0.05; ns, not significant).

The roles of NFAT5 played on BoHV-1 productive infection were analyzed in MDBK cells by knockdown of NFAT expression using specific siRNA. For this purpose, an effective siRNA targeting NFAT5 referred to as siRNANFAT5-3 was screened. It reduced NFAT5 protein levels to approximately 25% relative to the control (Fig. 6 A and B). The specific siRNANFAT5-3 increased virus production by approximately 1.96-fold relative to the scrambled siRNA when cell-associated viral genomes were examined by qPCR (Fig. 6C). The virus titers in the supernatant were increased to approximately 0.8-log by transfection of NFAT5-specific siRNA, relative to that of transfected scrambled siRNA controls (Fig. 6D). Thus, knockdown of NFAT5 expression by siRNA increases viral productive infection, suggesting that NFAT5 is a host factor that can restrict virus productive infection. When the NFAT5 plasmid that expresses human NFAT5 was transfected into MDBK cells, NFAT5 protein were overexpressed as expected (Fig. 6E). Consequently, the virus titer was decreased over 1-log by the transfection of either 1 or 2.5 μg of plasmid, respectively (Fig. 6F), which further validated the results of siRNA transfection.

**FIG 7:**
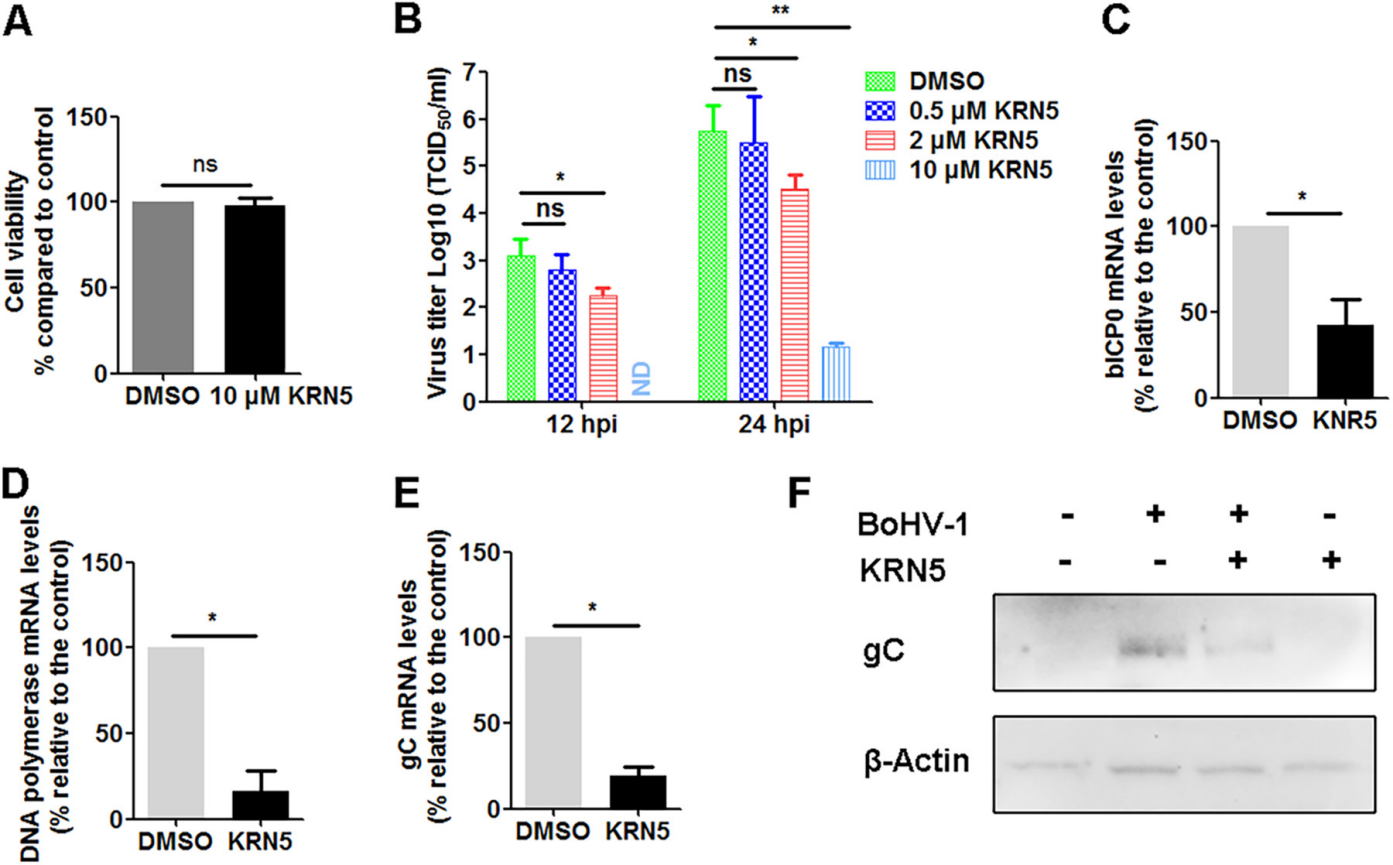
Effects of NFAT5-specific inhibitor KRN5 had on BoHV-1 productive infection. (A) MDBK cells in 24-well plates were treated with either DMSO control or 10 μM KRN5. After treatment for 24 h, the cytotoxicity in MDBK cells was analyzed with a Trypan-blue exclusion test. (B) Confluent MDBK cells in 24-well plates were pretreated with either DMSO control or KRN5 at a concentration ranging from 0.5 to 10 μM at 2 h before infection. The cells were infected with BoHV-1 at MOI of 0.1 for 1 h in the presence of either DMSO or KRN5. After extensive washing with PBS, a fresh medium containing inhibitors or DMSO control was replaced. At 12 and 24 h postinfection, the cell cultures were collected, and virus titers were measured with results expressed as TCID_50_/mL. (C to E) As described in panel B, MDBK were treated with 2 μM KRN5 during virus infection plus a pretreatment for 2 h prior to infection. After infection for 24 h, total RNA was isolated and analyzed by qRT-PCR to measure viral mRNA transcripts of bICP0 (C), DNA polymerase (D), and gC (E) by using relative qRT-PCR. (F) As described in panels C to E, MDBK cells in 6-well plates were either mock infected or infected with BoHV-1 along with treatment of either DMSO or 2 μM KRN5 during virus infection. After infection for 24 h, cell lysates were prepared and subjected to Western blot to detect viral protein gC. Data represent the means of three independent experiments. Significance was assessed with Student's *t* test (*, *P* < 0.05).

KRN5 is a chemical that inhibits NFAT5 activity without interrupting its downstream targets. As a potential therapeutic agent in the treatment of chronic arthritis, it shows stronger effects than the conventional used drug methotrexate (26). The effect that the NFAT5 signaling pathway has on virus productive infection was further examined using the chemical inhibitor KRN5. The effects of KRN5 at a concentration ranging from 0.5 to 10 μM on virus productive infection at two different times after infection were analyzed in MDBK cells. KRN5 at a concentration of 10 uM had no obvious toxic effects on MDBK cells (Fig. 7A), as determined by the trypan-blue exclusion test described by Fiorito, et al. (27). Relative to the DMSO control, 0.5 μM KRN5 had no obvious effects on virus replication at either 12 and 24 h postinfection, while 2 or 10 μM KRN5 consistently reduced the levels of infectious virus at both 12 and 24 h after infection (Fig. 7B). In comparison to the DMSO controls, 2 μM KRN5 decreased the virus yield approximately by 1.63- and 1.22-log after infection for 12 h. When the virus-infected cells were treated with 10 μM KRN5, the virus titer was rarely detected after infection for 12 h, suggesting a strong inhibitory efficiency. Relative to the vehicle DMSO, 10 μM KRN5 reduced the virus production by approximately 4.57-log (Fig. 7B). These data consistently suggested that KRN5 has the capacity to inhibit virus productive infection, which is different from observed with knockdown of NFAT5 via using siRNA.

**FIG 8:**
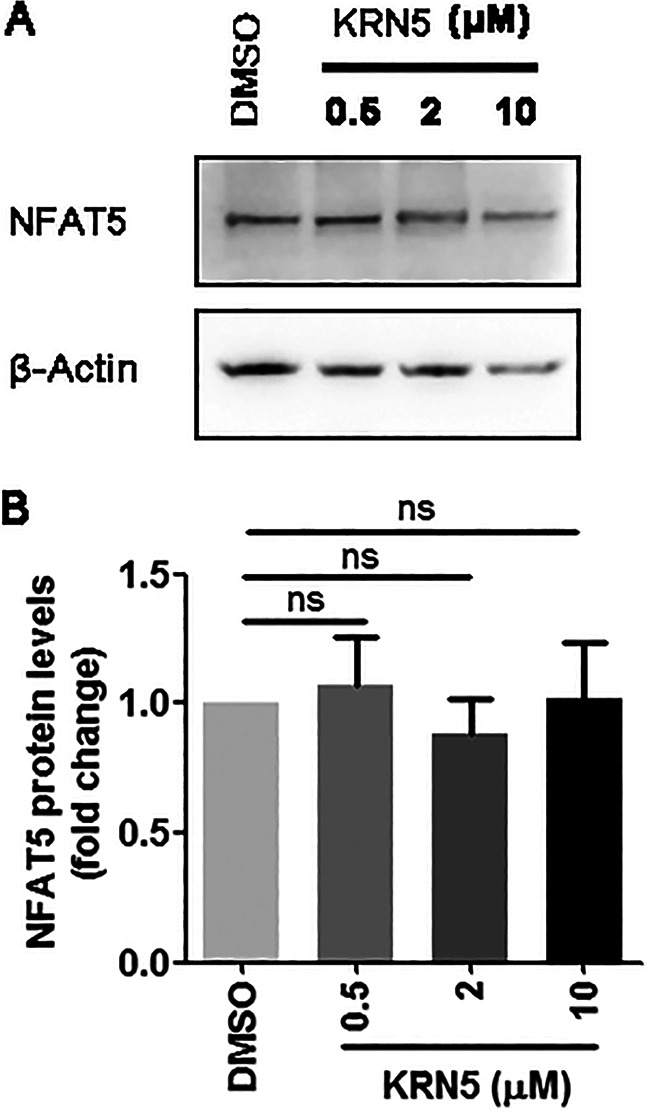
Effects of KRN5 had on NFAT5 protein expression in MDBK cells. (A) MDBK cells in 6-well plates were treated with either DMSO control or KRN5 at indicated concentrations. After treatment for 24 h, the cell lysates were prepared and subjected to Western blot to detect the protein levels of NFAT5. (B) Band intensity was analyzed with the software Image J. The control was arbitrarily set as 100%. Data represent the means of three independent experiments. Significance was assessed with Student's *t* test (ns, not significant).

Because NFAT5 is a transcriptional factor regulating transcription of its downstream targets, the effects that KRN5 had on mRNA expression of BoHV-1 immediate early (IE) proteins, such as the viral regulatory proteins (bICP0), viral DNA polymerase, and viral glycoprotein gC were examined in productively infected MDBK cells. As a result, mRNA levels of all the detected genes were decreased by treatment of 2 μM KRN5. The mRNA levels of bICP0 (Fig. 7C), viral DNA polymerase (Fig. 7D), and viral glycoprotein gC (Fig. 7E) were decreased to approximately 42.15%, 16.25%, and 19.20%, respectively. To validate the data of qPCR, the expression of gC protein affected by KRN5 treatment was detected by Western blotting. As expected, gC protein expression was significantly reduced by 2 μM KRN5. Of note, the treatment of MDBK cells with KRN5 has no effects on NFAT5 protein expression (Fig. 8).

Taking these data together, KRN5 significantly reduced virus productive infection partially via inhibition of viral gene expression, which does not support our findings that depletion of NFAT5 protein by siRNA leads to increased viral production. Off-target effects of KRN5 may account for the discrepancy.

## DISCUSSION

In addition to the pathogenesis of various inflammatory diseases and cancers, NFAT5 is involved in the induction of diseases by multiple viruses, such as Coxsackievirus B3 (CVB3) (28, 29), hepatitis B virus (HBV) (30), HCV (31), lymphocytic choriomeningitis virus (LCMV) (32), and human immunodeficiency virus-1 (HIV-1) (33, 34). To our knowledge, the involvement of NFAT5 in alphaherpesvirus infection has not been reported. Here, for the first time, we showed that NFAT5 is involved in BoHV-1 productive infection as virus infection relocalized NFAT5 and reduced accumulation of NAFT5 proteins in both cytoplasm and mitochondria (Fig. 2–4). Moreover, we found that the knockdown of NFAT5 protein expression by transfection of NFAT5-specific siRNA increased virus production (Fig. 6). Indeed, we do not know the mechanism regarding how BoHV-1 replication was limited by NFAT5, which is an interesting question that deserves an independent study in the future. We assume that the activation of innate immunity-related proteins by NFAT5 may account for the antiviral effects because, as a transcription factor, NFAT5 is implicated in regulating innate immunity and immune metabolism (5, 35). Similarly, it has been reported that NFAT5 inhibits CVB3 replication partially via enhancing expression of inducible nitric oxide synthase (iNOS) (29), providing a sample that supports our speculations. Interestingly, when starting to prepare our manuscript, one paper was published, which reported that NFAT5 haploinsufficiency may potentially correlate with Epstein-Barr virus (EBV, also called human gammaherpesvirus 4) susceptibility as observed in two patients (18), indicating that NFAT5 may restrict EBV infection *in vivo*, as observed in BoHV-1 productive infection *in vitro*. Together with these reports, antiviral effects of NFAT5 may universally apply to broad spectrum of viruses, including herpesvirus.

NFAT5-specific inhibitor, KRN5, is a potential drug with a strong capacity in the treatment of chronic arthritis (26). Surprisingly, KRN5 showed strong inhibitory effects on virus productive infection (Fig. 7B), which is inconsistent with the findings that depletion of NFAT5 by siRNA promotes virus production (Fig. 6). We speculated that off-targets effects of the chemical might account for the discrepancy. KRN5 is a chemical that has strong antiviral effects against BoHV-1 productive infection in cell cultures. Whether it also has antiviral effects *in vivo* is an interesting issue deserving further studies in animals.

Five different NFAT5 isotypes, including NFAT5a (158 kDa), NFAT5b (11 kDa), NFAT5c (166 kDa), NFAT5d1 (167.7 kDa), and NFAT5d2 (167.8 kDa), generated via alternative splicing have been described in human cells (36, 37). Among these five isoforms, a, b, and c translocate into the nucleus in response to osmotic stress (36). Meanwhile, the isoforms of NFAT5 in Bos taurus has not been reported, and the major NFAT5 bands with molecular weights of around 180 kDa were readily detected in MDBK cells of both fractions of nucleus and cytosol (Fig. 4). In addition to the major NFAT5 bands with a molecule size at around 180 kDa, another two bands with distinct molecular weights, including band A of more than 180 kDa, and band B of less than 180 kDa, were obtained (Fig. 4). It is highly possible that these different bands are distinct NFAT5 isoforms produced by alternative splicing. Of note, posttranslational modifications of NFAT5 play important roles in the regulation of NFAT5 enter nucleus, as well as its biological activates (38, 39). For example, the palmitoylation and phosphorylation of NFAT5c is required to translocate into the nucleus (40). Therefore, it is also possible that the band A is produced partially due to posttranslational modifications. BoHV-1-encoded viral proteins, such as bovine herpesvirus 1-infected cell protein 0 (bICP0), promote the proteasome-mediated degradation of certain cellular proteins, including interferon response factor 3 (IRF3) and promyelocytic leukemia (PML) (41, 42). Therefore, the referred NFAT5 band B may be resulted from proteolytic cleavage of full-length NFAT5. We noticed that both band A and band B were exclusively observed in the nucleus rather than the cytoplasm of both mock-infection and virus-infected cells. Thus, virus productive infection has differential effects on the nuclear accumulation of these two isoforms or molecules (Fig. 4). Of course, we could not exclude the possibility that the bands A and B are nonspecific binding of the antibody to other nuclear proteins. The mRNA levels of the canonical downstream targets regulated by NFAT5 signaling, including PGK1, SMIT, and BGT-1, were differentially altered by virus infection with unexpected manners (Fig. 4). Though isoforms of NFAT5 a, b, and c have capacity to translocate into the nucleus, NFAT5 isoform-specific regulation of the transcription of the downstream targets are largely unknown. We could not precisely delineate whether these distinct NFAT5 bands are associated with the expression of these downstream targets. Thus, it is an interesting question to reveal the mechanism of why and how these isoforms or molecules were produced, as well as the implications in the virus productive infection.

It has been reported that the transcription factor NFAT5 contributes to the glycolytic phenotype by regulating the downstream targets phosphoglycerate kinase 1 (PGK-1), which is the first enzyme generating ATP in the glycolysis (43), indicating that NFAT5 participates in the regulation of glycolysis. In addition, NFAT5 also participates in oxidative phosphorylation (OXPHOS) because hypoxia-exposed Nfat5-deficient pulmonary artery elevated the levels of OXPHOS-related transcripts and mitochondrial respiration, indicating that NFAT5 contributes negatively to OXPHOS in mitochondria (44). BoHV-1 infection affected the expression of certain components in mitochondrial respiration complexes, such as SDHB (succinate dehydrogenase) for complex II and cytochrome c oxidase subunit I (MTCO1) (45). Whether NFAT5 is involved in the alteration of these components remains to be determined in the future. Interestingly, for the first time, we found that NFAT5 resides in mitochondria, a critical place where glycolysis and OXPHOS mainly occurred, which provides a novel aspect to explain how mitochondrial functions were affected by NFAT5, which deserves further studies in the future.

In summary, these studies indicated that virus productive infection at late stages of may have effects on NFAT5 signaling pathway via relocalization of NFAT5 proteins and changed accumulation of NFAT5 in distinct subcellular fractions. And for the first time, we showed that a subset of NFAT5 molecules resides in mitochondria, providing an evidence to regulate mitochondrial functions. In addition, we found that NFAT5 is a host factor limiting the virus’s productive infection.

## MATERIALS AND METHODS

### Cells and viruses.

MDBK (purchased from Chinese model culture preservation center, Shanghai, China) were routinely passaged and cultured in DMEM containing 10% fetal bovine serum (FBS). BoHV-1 stain NJ-16-1 (46) was propagated in MDBK cells. Aliquots of virus stocks were stored at −70°C until use.

### Antibodies and reagents.

The following antibodies were used in this study: NFAT5 monoclonal antibody (MAb) (cat# sc-398171) and LaminA/C mouse MAb (cat# sc-376248) were provided by Santa Cruz Biotechnology (Dallas, TX, USA). HSP60 rabbit polyclonal antibody (pAb) (cat#A0969) and β-Tubulin rabbit pAb (cat# AC015) were obtained from Abclonal (Woburn, MA, USA). β-actin rabbit MAb (cat# 4970), HRP conjugated goat anti-mouse IgG (cat# 7076), as well as HRP, labeled goat anti-rabbit IgG (cat# 7074) were purchased from Cell Signaling Technology (Danvers, MA, USA). BoHV-1 gC MAb (cat#F2) was provided by VMRD Inc. (Pullman, WA, USA). Alexa Fluor 633-conjugated goat pAb to mouse IgG (cat#A-21052) was provided by Invitrogen Life Technologies (Waltham, MA, USA).

### Western blotting analysis.

Cell lysates of either whole-cell extracts or cellular fractions, including cytosol, nucleus, and mitochondria, were prepared using RIPA lysis buffer (1 × PBS, 1% NP-40, 0.5% sodium deoxycholate, 0.1% SDS) supplemented with protease inhibitor cocktail. They were boiled in Laemmli sample buffer for 5 min, and subsequently subjected to separation on SDS-PAGE (8% or 10%), and then transferred to polyvinylidene fluoride (PVDF) membranes. Immuno-reactive bands were developed using Clarity Western ECL Substrate (Bio-Rad, cat# 1705061). Western blots were repeated at least three times for each study.

### siRNA and plasmid transfection assay.

MDBK cells in 6-well plates were transfected with scrambled siRNA or three NFAT5 specific siRNA of 200 pmol, provided by Genepharma (Shanghai, China) and plasmid pEGFP-NFAT5 (no EGFP), by using Lipofectamine 3000 (Invitrogen). At 48 h posttransfection, cell lysates were prepared by using cell lysis buffer as described above, and subjected to Western blot to detect the protein levels of NFAT5, or the cells were infected by BoHV-1 (MOI = 0.1) for 24 h. Progeny virus were detected in MDBK cells, with results expressed as TCID_50_/mL calculated using the Reed-Muench formula. The plasmid pEGFP-NFAT5 (no EGFP) was a gift from Anjana Rao (Addgene plasmid # 13627) (7).

### Immunofluorescence assay.

MDBK cells in 8-well chamber slides (Nunc Inc., IL, USA) were mock infected or infected with BoHV-1 (MOI = 0.1). After infection for 24 h, cells were fixed with 4% paraformaldehyde in PBS for 10 min at room temperature, permeabilized with 0.25% Triton X-100 in PBS for 10 min at room temperature, and blocked with 1% BSA in PBST for 1 h followed by incubation with NFAT5 MAb (1:300) in 1% BSA in PBST overnight at 4°C. After three washings; cells were incubated with secondary antibody labeled with distinct fluorescent dyes for 1 h in the dark at room temperature. After three washings, DAPI (4r,6-diamidino-2-phenylindole) staining was performed to visualize nuclei. Slides were covered with coverslips using an antifade mounting medium (Electron Microscopy Sciences, cat# 50-247-04). Images were captured using a confocal microscope (Leica).

### RNA isolation and quantification of mRNA by qRT-PCR.

Total RNA was extracted from infected cells or uninfected control cells using a TRIzol LS reagent (Ambion, cat# 10296010) following the manufacturer's instructions. Freshly prepared total RNA (1 μg) was used as a template for synthesizing first-strand cDNA with commercial random hexamer primers using Thermoscript RT-PCR system Kit (Invitrogen, cat# 11146-024) following the manufacturer's instructions. The cDNA products were then used as templates for real-time quantitative PCR to measure mRNA levels of indicated genes with gene-specific primers. For these studies, we analyzed the betaine-γ-amino-butyric acid transporter (BGT-1) (forward primer 5′-CTGGGAGAGACGGGTTTTGGGTATTACATC-3′ and reverse primer 5′-GGACCCCAGGTCGTGGAT-3′), the sodium-dependent myo-inositol cotransporter (SMIT), (forward primer 5′-CCGGGCGCTCTATGACCTGGG-3′ and reverse primer 5′-CAAACAGAGAGGCACCAATCG-3′) (25), phosphoglycerate kinase 1 (PGK-1) (forward primer 5′-GAACAAGGTTAAAGCCGAGCC-3′ and reverse primer: 5′-GTGGCAGATTG-ACTCCTACCA-3′), NFAT5 (forward primer 5′-GGGTCAAACGACGAGATTGTG-3′ and reverse primer: 5′-GTCCGTGGTAAGCTGAGAAAG-3′) (43), gC(forward primer 5′-ACTATATTTTCCCTTCGCCCG-3′ and reverse primer: 5′-TGTGACTTGGTGCCCATG-3′), and BoHV-1 DNA polymerase (forward primer 5′-GCGAGTACTGCATCCAAGAT-3′ and reverse primer: 5′-AATCTGCTGCCCGTCAAA-3′). Primers used to detect bICP0, bICP22, and GAPDH were described elsewhere (47).

Analysis of glyceraldehyde-3-phosphate dehydrogenase (GAPDH) mRNA was used as an internal control. Real-time PCR was carried out using the ABI 7500 fast real-time system (Applied Biosystems, CA). The expression levels of the tested genes were normalized to that of the GAPDH gene. The relative mRNA level of each gene was calculated using the method (2^-ΔΔCT^) by comparison to the control.

### DNA purification and quantification of viral DNA by qPCR.

Total genomic DNA was extracted from infected cells via using a commercial viral DNA purification kit (Tiangen, cat# DP-348) following the manufacturer's instructions. Freshly prepared DNA was used as a template for real-time qPCR to measure viral DNA levels with gC-specific primers, as described above.

Analysis of GAPDH was used as an internal control. Real-time PCR was carried out using the ABI 7500 fast real-time system (Applied Biosystems, CA). The levels of viral genome represented by gC were normalized to that of the GAPDH gene. The relative levels of the vial genome were calculated using the method (2^-ΔΔCT^) by comparison to the control.

### KRN5 treatment of MDBK cells during virus infection.

MDBK cells of confluent in 24-well plates were infected with BoHV-1 (MOI = 0.1) along with the treatment of chemical KRN5 (MCE, cat# HY-112126) at the indicated concentration for 1 h at 37°C. After three washing with PBS, fresh medium with KRN5 at indicated concentrations was added to each well. After infection for 12 and 24 h, viral yields were titrated in MDBK cells, respectively. Cell cultures treated with DMSO was used as a control. The results were expressed as TCID50/mL calculated using the Reed-Muench formula.
